# Ewing's Sarcoma of the Frontal Bone with Intracranial Extension

**DOI:** 10.1055/a-2649-0851

**Published:** 2025-07-14

**Authors:** Sana Ahuja, Shaivy Malik, Charanjeet Ahluwalia

**Affiliations:** 1 Department of Pathology, Vardhman Mahavir Medical College and Safdarjung Hospital, New Delhi, India

**Keywords:** Ewing's sarcoma, frontal bone, intracranial, bone tumor

## Abstract

Ewing's sarcoma, though rare, primarily affects children and young adults, commonly manifesting in long bones. Cranial involvement, particularly in the frontal bone, is exceptionally uncommon, posing diagnostic and therapeutic challenges. Meticulous pathological assessment is crucial for recognizing and managing such atypical presentations. A 16-year-old male presented with left frontal swelling and neurological symptoms. Imaging revealed a space-occupying lesion involving the left frontal bone with intracranial extension. Histopathology confirmed Ewing's sarcoma based on characteristic findings and positive immunohistochemical markers. Differential diagnoses include metastatic neuroblastoma, primitive neuroectodermal tumors, and chordomas, highlighting the importance of comprehensive evaluation. Ewing's sarcoma involving the skull necessitates a multidisciplinary approach for accurate diagnosis and management. This case underscores the significance of clinical, radiological, and pathological assessments in recognizing rare manifestations. Collaboration among teams is crucial for tailored management strategies and optimal patient outcomes.

## Introduction

Ewing's sarcoma is a rare and aggressive primary bone tumor that primarily affects children and young adults. It most commonly arises in the long bones of the extremities, pelvis, and chest wall. However, involvement of the cranial bones is exceedingly rare, accounting for less than 1% of all cases with the temporal bone being most frequently involved. 1 Furthermore, intracranial extension of Ewing's sarcoma is exceptionally uncommon, presenting unique diagnostic and therapeutic challenges. By elucidating this unusual pathological manifestation, we underscore the importance of meticulous pathological assessment in recognizing and managing atypical presentations of Ewing's sarcoma. Comprehensive pathological evaluation, including histopathology and immunohistochemistry, was pivotal in establishing the diagnosis. Herein, we report a case of Ewing's sarcoma involving the frontal bone with intracranial extension in a 16-year-old male.

## Case Presentation

**Fig. 1:**
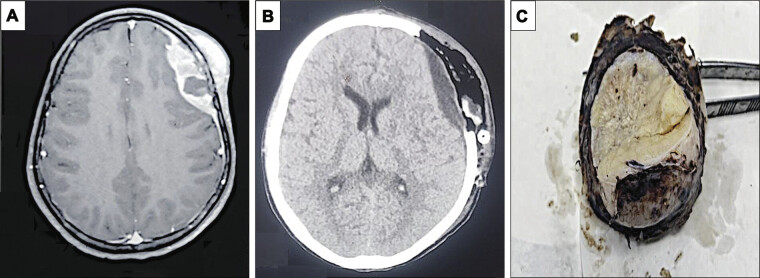
Radiological and gross findings. ( **A** ) Magnetic resonance imaging revealed a 5.1 × 1.8 cm hetero-intense space-occupying lesion involving the left frontal bone with an overlying soft tissue component and intracranial extension. ( **B** ) Computed tomography showed a well-defined space-occupying lesion in the left frontal region involving the left frontal bone with associated overlying soft tissue component. ( **C** ) Gross findings exhibited a part of the frontal bone along with attached fleshy tan-white tumor measuring 5 × 2 cm.

A 16-year-old male presented to our hospital with complaints of left frontal swelling along with progressively worsening headaches, nausea, and intermittent episodes of vomiting over the past 3 months. On examination, he was found to have bilateral papilledema, which was graded as Frisen grade II on fundoscopy. No additional visual disturbances such as blurring, diplopia, or visual field defects were reported. The presence of bilateral papilledema despite a relatively small lesion with minimal midline shift and open cortical sulci was unexpected. We hypothesize that this may be attributed to intermittent rises in intracranial pressure due to compromised venous outflow or dynamic shifts in cerebrospinal fluid (CSF) pressure, possibly exacerbated by the lesion's frontal location and compressive effect on adjacent brain parenchyma. Neurological examination revealed no focal deficits. Initially, magnetic resonance imaging (MRI) was advised, and it revealed an approximately 5.1 × 1.8 cm hetero-intense space-occupying lesion involving the left frontal bone with an overlying soft tissue component and intracranial extension that appeared confined to the epidural compartment, causing compression of the left frontal lobe. Computed tomography showed a well-defined space-occupying lesion in the left frontal region involving the left frontal bone with associated overlying soft tissue component ( Fig. 1A, B ). A differential diagnosis of primary neoplasm versus metastasis was suggested based on the radiological findings. A frontotemporal craniectomy was performed followed by gross total excision of the tumor with overlying bone and sent for histopathological examination.

**Fig. 2:**
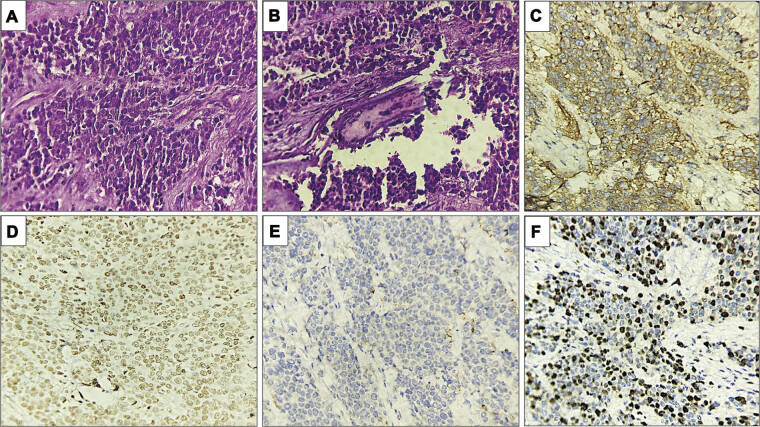
Histopathological and immunohistochemical findings. ( **A** ) Hematoxylin and eosin–stained sections examined showed a monomorphic population of small round cells arranged in trabeculae and lobules with intervening connective tissue septae (400x). ( **B** ) The tumor cells were seen invading the bony fragments (400x). ( **C–F** ) Immunohistochemistry exhibited positive immunostaining for CD99 ( **C** ) and FLI-1 ( **D** ) with negative staining for glial fibrillary acidic protein (GFAP) ( **E** ). A K _i_ -67 proliferation index of 50% was seen (400x).

An excision specimen of a part of the frontal bone along with an attached fleshy tan-white tumor measuring 5 × 2 cm was received ( Fig. 1C ). The tumor was partly encapsulated and was seen breaching the bone. Hematoxylin and eosin–stained sections examined showed a monomorphic population of small round cells arranged in trabaculae, lobules, and microfollicles with intervening connective tissue septae. Individual tumor cells had scant cytoplasm, round nuclei with stippled chromatin, and inconspicuous nucleoli. Focal areas of necrosis with brisk mitosis (>20/10 high power fields) were also seen. Immunohistochemistry was performed and tumor cells exhibited positive expression for CD99 (rabbit monoclonal, clone EP8, Biocare Medical), vimentin (mouse monoclonal, clone V9, Biocare Medical), and FLI-1 (mouse monoclonal, clone G146–222, BioSB) while being negative for glial fibrillary acidic protein (GFAP) (mouse monoclonal, clone GA-5, Biocare Medical) and synaptophysin (mouse monoclonal, clone 27G12, Biocare Medical). The K _i_ -67 labeling index (mouse monoclonal, clone MIB-1, Biocare Medical) was around 50% ( Fig. 2 ). Based on the above findings, a diagnosis of Ewing's sarcoma was made. Fluorescent in situ hybridization was done which showed the presence of EWS gene translocation confirming the diagnosis. Positron emission tomography (PET) scan was performed, and no extracranial tumor was identified. Further, there were no signs or symptoms suggestive of an extracranial tumor anywhere else.

## Discussion

Ewing's sarcoma is a rare occurrence in the skull, accounting for less than 1% of cases. It typically affects the temporal, parietal, and less commonly the frontal bone. 1 Typical symptoms at the time of diagnosis often include local swelling, as observed in our case, along with associated headaches. Patients with the primary site of the skull base may exhibit proptosis and various cranial nerve palsies. 2 Intracranial extension or involvement of neural structures may lead to symptoms of raised intracranial pressure and focal neurological deficits, which were present in the present case.

Accurate diagnosis of Ewing's sarcoma in the skull requires a meticulous histopathological approach, as its small round blue cell morphology can closely resemble several other malignant entities. Without thorough evaluation, including extensive immunohistochemistry and molecular confirmation, there is a risk of misdiagnosis as lymphoma, neuroblastoma, rhabdomyosarcoma, or other primitive neuroectodermal tumors. The diagnosis is particularly challenging when the tumor presents in uncommon locations like the skull, where primary bone malignancies are rare. 3 Histologically, the uniform appearance of small round cells with scant cytoplasm may lack distinguishing features, and thus reliance on CD99 positivity alone is insufficient. Therefore, we employed a comprehensive panel including CD99, FLI-1, and vimentin, and confirmed the diagnosis through EWS gene translocation by FISH, which we consider essential to reduce diagnostic uncertainty.

One of the primary considerations in the differential diagnosis of Ewing's sarcoma at this site is metastatic neuroblastoma, especially in pediatric patients with skull tumors. This aggressive malignancy originating from neural crest cells may metastasize to the skull, leading to localized swelling or pain. Primitive neuroectodermal tumors (PNETs) also warrant attention, given their histological similarities with Ewing's sarcoma. 4 5 Discriminating between these entities relies on immunohistochemical analysis, which helps delineate their distinct molecular profiles as they express neuronal markers like neuron-specific enolase and synaptophysin.

Chordomas, arising from notochord remnants, present another diagnostic challenge, often mirroring the symptoms of Ewing's sarcoma such as local pain and swelling. Immunohistochemical staining for specific markers (brachyury) aids in the differentiation process. Furthermore, lymphoma, whether primary or secondary, can involve the skull and manifest with swelling or localized pain. Accurate diagnosis requires meticulous histopathological examination and immunohistochemistry. 4 5

Rhabdomyosarcoma, osteosarcoma, and meningioma are additional entities that should be considered in the differential diagnosis. Imaging studies and histopathological examination play critical roles in distinguishing these conditions.

Moreover, desmoplastic small round cell tumors (DSRCTs), plasmacytomas, and solitary metastases merit inclusion in the differential diagnosis. DSRCTs, though rare, can metastasize to the skull and typically present with abdominal masses and symptoms related to metastasis. Plasmacytomas, originating from plasma cells, may present with localized pain and swelling in the skull. Solitary metastases from other primary sites necessitate careful consideration of the patient's clinical history and imaging findings. 4

In our experience, the lack of standardized diagnostic algorithms in skull-based Ewing's sarcomas leads to potential diagnostic delays or inaccuracies. A literature review reveals that in some cases, definitive diagnosis is achieved only after resection or on referral to tertiary centers with molecular diagnostic facilities. 3 Our case reinforces the importance of integrating radiological, histopathological, immunohistochemical, and molecular features in a stepwise and coordinated manner, which we consider to constitute the “meticulous” approach referenced throughout the manuscript.

Table 1 summarizes the clinicopathological and radiological findings of the previously reported cases of Ewing's sarcoma of the frontal bone. Compared with previously reported cases summarized in Table 1 , our case presents several distinctive features. In contrast to the cases by Sharma et al and Gupta et al, where local swelling was the predominant complaint, our patient exhibited persistent symptoms of raised intracranial pressure such as severe headaches, nausea, and vomiting, lasting for 3 months. 4 5 Although Mohindra et al also documented such symptoms in several patients, our case differs in terms of the lesion's anatomical involvement—demonstrating focal compression of the left frontal lobe. 8 However, we acknowledge that the intracranial extension in the case reported by Gupta et al 4 included a large epidural component, and thus the assertion that our case represents a more extensive intracranial disease should be revised.

**Table 1:** Clinicopathological and radiological features of previously reported cases of Ewing's sarcoma of the frontal bone

Author	Age/ Gender	Clinical presentation	Radiological findings	Immunohistochemical profile	Treatment	Follow-up
Gupta et al (2012) 4	11/F	Painless swelling in left frontoparietal region from 8 months	MRI: A lytic lesion in left frontoparietal calvarium with sun ray periosteal reaction, with a subgaleal and epidural component causing bulking of parenchyma	Positive for MIC-2	Surgical excision	No evidence of recurrence/ metastasis
Sharma et al (2017) 5	13/M	A painless lump over the left side of the head for 2 years	MRI: A well-defined space-occupying lesion of left frontal bone	Positive for vimentin, CD99, and FLI1 while negative for synaptophysin, glial fibrillary acidic protein (GFAP)	Surgery followed by adjuvant chemotherapy and local radiotherapy	–
Agrawal et al (2009) 6	11/M	A painful swelling over the right frontal region for 3 months	CT: An isodense lesion with bony spicule formation with intra- and extracranial extension	–	Surgery followed by adjuvant chemotherapy and local radiotherapy	No evidence of recurrence
Misra et al (2019) 7	17/M	Frontal swelling for 6 months duration	CT: An enhancing osteolytic soft tissue mass in the frontoparietal area	–	Surgery followed by adjuvant chemotherapy and local radiotherapy	No evidence of recurrence/ metastasis
Mohindra et al (2022) 8	14/M	Raised intracranial pressure, vision loss	MRI: Extra + intradural, Gd-enhancing variegated, solid, cystic	–	Surgery, chemotherapy, and radiotherapy	No evidence of recurrence/ metastasis
	19/F	Raised intracranial pressure, tinnitus	MRI: Extracranial, extradural, Gd-enhancing variegated, solid	–	Surgery, chemotherapy and radiotherapy	No evidence of recurrence/ metastasis
	2/M	Raised intracranial pressure, neck pain	MRI: Extracranial, extra + intradural, Gd-enhancing variegated, solid, cystic	–	Shunt, excision, radiotherapy	Death at 6 months
	8/M	Raised intracranial pressure, swelling	MRI: Extracranial, extradural, Gd-enhancing variegated, solid	–	Excision, chemotherapy, radiotherapy	No evidence of recurrence/ metastasis
	17/F	Raised intracranial pressure, hemiparesis	MRI: Extracranial, extra + intradural, intraorbital, Gd-enhancing, solid, cystic	–	Excision, chemotherapy, radiotherapy	Death at 3 years
	14/F	Swelling, skin erosion	MRI: Extracranial, Gd-enhancing variegated, solid	–	Excision, chemotherapy, radiotherapy	No evidence of recurrence/ metastasis
	17/M	Raised intracranial pressure, swelling	MRI: Extracranial, extradural, Gd-enhancing variegated, solid	–	Excision, chemotherapy, radiotherapy	No evidence of recurrence/ metastasis
	6/M	Failure to thrive	MRI: Intracranial, extradural, Gd-enhancing variegated, solid	–	Excision, chemotherapy	No evidence of recurrence/ metastasis
Present case	16/M	Left frontal swelling along with worsening headaches, nausea, and intermittent episodes of vomiting for 3 months	MRI: A 5.1 × 1.8 cm hetero-intense space-occupying lesion involving the left frontal bone with an overlying soft tissue component and intracranial extension	Positive for CD99, FLI-1, vimentin and negative for GFAP, synaptophysin	Surgery followed by adjuvant chemotherapy	No evidence of recurrence/ metastasis

We have amended our comparison to reflect that although our case demonstrates a typical pattern of raised intracranial pressure and intracranial extension, it does not significantly differ in radiological extent from some cases described by Mohindra et al and Gupta et al. 4 8 Additionally, the comprehensive pathological evaluation, including immunohistochemistry and molecular studies, further contributes to the novelty of this report, providing valuable insights for the pathology community.

Radiographic findings commonly include areas of bone destruction with ill-defined margins on plain radiographs. Computed tomography scans exhibit an isodense lesion with significant heterogeneous enhancement. MRI is particularly valuable in delineating soft tissue involvement, distinguishing Ewing's sarcoma from other skull-based lesions, including metastatic tumors or other primary bone malignancies. MRI's superior contrast resolution helps in identifying the degree of soft tissue extension and neural involvement, which is essential for treatment planning. Biopsy and immunohistochemistry are crucial for a definitive diagnosis. 4 5 6 7

The current standard of care for Ewing's sarcoma, including calvarial lesions, involves a multimodal treatment approach. This typically includes neoadjuvant chemotherapy to reduce tumor burden, followed by surgical resection when feasible, and adjuvant local therapy with radiotherapy. Postoperative chemotherapy is essential to eradicate residual microscopic disease. In cases involving the skull, especially with intracranial extension, precise radiotherapy planning becomes crucial to minimize damage to surrounding neural structures. Advances in imaging, particularly MRI, also guide the extent of surgery and radiotherapy fields, contributing to improved outcomes. 7 8 In our case, the patient underwent complete surgical excision, followed by adjuvant chemotherapy. Although radiotherapy was not administered due to favorable surgical margins and clinical parameters, it remains a critical component in standard treatment protocols, especially when gross total resection is not achieved or residual disease is suspected.

Although Ewing's sarcoma involving the skull remains a rare occurrence, a comprehensive understanding of its differential diagnosis is essential. Accurate diagnosis relies on a multifaceted approach, including clinical evaluation, radiological imaging, histopathological examination, and immunohistochemistry. By documenting this unique presentation, the authors aim to enrich the existing literature on rare manifestations of Ewing's sarcoma. Collaboration among multidisciplinary teams is imperative to ensure timely and tailored management strategies for optimal patient outcomes.

## References

[1] Rehman R Osto M Parry N Ewing sarcoma of the craniofacial bones: a qualitative systematic review Otolaryngol Head Neck Surg 2022 166 04 608 614 34255595 10.1177/01945998211022228

[2] Zare F Shahbazi N Faraji N Goli R Mostafaei B Anari S A cruel invasion of Ewing's sarcoma of the skull: a rare case report Int J Surg Case Rep 2023 108 108380 37406533 10.1016/j.ijscr.2023.108380 PMC10382727

[3] Trikalinos N A Chrisinger J SA Van Tine B A Common pitfalls in Ewing sarcoma and desmoplastic small round cell tumor diagnosis seen in a study of 115 cases Med Sci (Basel) 2021 9 04 62 34698236 10.3390/medsci9040062 PMC8544526

[4] Gupta A Bansal S Chaturvedi S Primary Ewing's sarcoma of frontoparietal bone with major soft tissue extension: an unusual presentation and review of the literature Case Rep Pathol 2012 2012 713836 23198232 10.1155/2012/713836 PMC3502810

[5] Sharma A D Singh J Bhattacharya J Primary Ewing's sarcoma of cranium in a pediatric patient J Pediatr Neurosci 2017 12 03 273 275 29204207 10.4103/jpn.JPN_29_17 PMC5696669

[6] Agrawal A Dulani R Mahadevan A Vagaha S J Vagha J Shankar S K Primary Ewing's sarcoma of the frontal bone with intracranial extension J Cancer Res Ther 2009 5 03 208 209 19841565 10.4103/0973-1482.57129

[7] Misra R Kumar S Sinha R Sharma S Ewing's sarcoma of frontal bone: a rare case Indian J Neurosurg 2019 8 139 141

[8] Mohindra S Tripathi M Batish A Primary calvarial Ewing sarcoma: a case series J Neurol Surg B Skull Base 2021 83 02 e181 e190 35832963 10.1055/s-0041-1722900 PMC9272254

